# Application of supervised machine learning algorithms for classification and prediction of type-2 diabetes disease status in Afar regional state, Northeastern Ethiopia 2021

**DOI:** 10.1038/s41598-023-34906-1

**Published:** 2023-05-13

**Authors:** Oumer Abdulkadir Ebrahim, Getachew Derbew

**Affiliations:** 1 Department of Public Health, College of Medical and Health Science, Samara University, Samara, Ethiopia; 2 College of Veterinary Medicine, Samara University, Samara, Ethiopia

**Keywords:** Health care, Medical research, Molecular medicine

## Abstract

Ethiopia has been challenged by the growing magnitude of diabetes in general and type-2 diabetes in particular. Knowledge extraction from stored dataset can be an important base for better decision on diabetes rapid diagnosis, suggestive on prediction for early intervention. Thus, this study was addressed these problem by application of supervised machine learning algorithms for classification and prediction of type 2 diabetes disease status and might provide context-specific information to program planners and policy makers so that, priority will be given to the more affected groups. To apply supervised machine learning algorithms; compare these algorithms and select the best algorithm based on their performance for classification and prediction of type-2 diabetic disease status (positive or negative) in public hospitals of Afar regional state, Northeastern Ethiopia. This study was conducted at Afar regional state from February to June of 2021. Decision tree; pruned J 48, Artificial neural network, K-nearest neighbor, Support vector machine, Binary logistic regression, Random forest, and Naïve Bayes supervised machine learning algorithms were applied using secondary data from the medical database record review. A total of 2239 sample Dataset diagnosed for diabetes from 2012 to April 22/2020 (1523 with type-2 diabetes and 716 without type-2 diabetes) was checked for its completeness prior to analysis. For all algorithms, WEKA3.7 tool was used for analysis purposes. Moreover, all algorithms were compared based on their correctly classification rate, kappa statistics, confusion matrix, area under the curve, sensitivity, and specificity. From the seven major supervised machine learning algorithms, the best classification and prediction results were obtained from random forest [correctly classified rate (93.8%), kappa statistics (0.85), sensitivity (0.98), area under the curve (0.97) and confusion matrix (out of 454 actual positive prediction for 446)] which was followed by decision tree pruned J 48 [correctly classified rate (91.8%), kappa statistics (0.80), sensitivity (0.96), area under the curve (0.91) and confusion matrices (out of 454 actual positive prediction for 438)] and k-nearest neighbor [correctly classified rate (89.8%), kappa statistics (0.76), sensitivity (0.92), area under the curve (0.88) and confusion matrices (out of 454 actual positive prediction for 421)]. Random forest, Decision tree pruned J48 and k-nearest neighbor algorithms have better classification and prediction performance for classifying and predicting type-2 diabetes disease status. Therefore, based on this performance, random forest algorithm can be judged as suggestive and supportive for clinicians at the time of type-2 diabetes diagnosis.

## Introduction

The world is overwhelming on an unprecedented rise in non-communicable diseases (NCDs) driven by urbanization, globalization of markets, and increasing longevity. Four major NCDs: diabetes, cardiovascular diseases, cancer and chronic respiratory diseases; are responsible for over 80% of NCD deaths of which more than 40% are premature, and the rest will occur in people under 70 years of age which is the productive age of the population^1,2^.

Diabetes is a chronic disease that occurs when the pancreas does not produce enough insulin (a hormone that regulates blood sugar) alternatively; when the body cannot effectively use the insulin it produces. The overall risk of dying among people with diabetes is at least double the risk of their peers without diabetes. Globally, this disease represents one of the major health and development challenges of the twenty-first century^3^.

In 2019, the international diabetes federation (IDF) also estimated that, in Africa (low and middle income countries), 19 million adults aged 20–79 years had diabetes, representing a prevalence of 5.2%. This region has also the highest proportion of previously undiagnosed diabetes; over two-thirds (67%) of people with diabetes being unaware they have the disease^4^.Currently, Ethiopia has been challenged by the growing magnitude of NCDs in general and diabetes in particular. Ethiopia is among the top four countries with the highest adult diabetic populations in sub-Saharan Africa. In 2019, the number of cases of this disease was 12,839,500 with a point prevalence of 10.2%^5,6^.

Patient attendance rates and medical admissions related to type-2 diabetes in major hospitals have been rising. This huge amount of data cannot be approached using the ordinal statistical methods to extract inferential information from it rather it needs a strong, complicated, and advanced model^7^. Rather, it requires a shift in healthcare priorities and up-to date data on the prediction and classification of type-2 diabetic disease status in Ethiopia, partially to help plan and priorities for health programmers. Such information can be an important base for policy on diabetes rapid diagnosis, real prediction for early prevention and treatment^6^.

There is a strong motivation and demand in the use of machine learning methods in knowledge discovery and data mining to generate models of health-related implications. Data Mining is about explaining the past and predicting the future. It is a collaborative field which combines technologies like statistics and informatics (machine learning, artificial intelligence, and database). The health care organizations are then able to use the extracted knowledge for more clients, disease diagnosis, classification, and prediction^8^.

This study on data mining (application of supervised machine learning algorithms) for classification and prediction of type-2 diabetic disease status partially might have a significant contribution for physicians and clients. For physicians it will help to facilitate diagnostic activity with suggestive and informative diagnostic results. On the other hand, for clients, an early diagnosis with accurate results will lead to early intervention. As a result, it will avoid unwanted catastrophic diagnostic cost and immediate management for prevention of morbidity and further complications of type-2 diabetes. Therefore, the main aim of this study was to extract hidden and critical knowledge by applying supervised machine learning algorithms for classification and prediction of type-2 diabetic disease status in public hospitals of Afar regional state Northeastern Ethiopia 2021.

## Materials and methods

### Study area and period

This study was conducted at public hospitals of Afar regional state Northeastern Ethiopia from February to June of 2021. Afar regional state is one of the nine federal states of Ethiopia located in the Northeastern part of the country 588 kms from Addis Ababa. The altitude of the region ranges from 1500 m above mean sea level (m.a.s.l) in the western highlands to − 120 m.a.s.l in the Danakil/Dallol depression. The total geographical area of the region is about 270,000 km^2^ and is geographically located between 39^o^34′ and 42^o^28′ East Longitude and 8^o^49′ and 14^o^30′ North Latitude (CSA, 2008). It has an estimated population of 1.2 million, of which, 90% are pastoralists (56% male and 44% female) and 10% are agro-pastoralists.

### Study design

A retrospective cross-sectional study design using medical database and medical chart record review was used.

### Target population

All hospital clients who ever diagnosed or will be diagnosed and/or suspected for type-2 diabetes in public hospitals of Afar regional state.

### Study population

All clients who ever diagnosed for diabetes disease status, and confirmed as free from type-2 diabetes (normal) and type-2 diabetic patient in public hospitals of Afar region starting from the year when the database for electronic health information record was fully functional up to the date of sample collection (2012 GC–April 22/2020) were considered as part of the study population.

### Eligibility criteria

#### Inclusion criteria

All clients who ever diagnosed for diabetes disease status and confirmed as free from type-2 diabetes (normal) and type-2 diabetic patients in public hospitals of Afar region starting from 2012 GC to April 22/2020 GC.

#### Exclusion criteria

Patients who can unable to obtain required information due to incomplete or total absence of their record status; which cannot be found in their registration book, diabetic patient under follow-up with unknown start date and diabetic patient referred from other hospitals were excluded from the study population to avoid misleading of the machine learning algorithms.

### Sample size determination, sampling method and procedure

All patients who have been diagnosed for diabetes and confirmed as a type-2 diabetic patient and normal after standard diagnostic activities from 2012 GC up to April 22/2020 GC were used as a sample. The whole dataset used as a sample because data mining needs considerable amount of data for effective prediction and classification by reducing the probability of error to be committed^9^. Based on this, the study has been conducted on a total of 2239 population.

From this record, the clients who ever diagnosed for diabetes disease status were extracted and collected with their required variables and some variables which are not available in the database and the parameters for the normal clients were searched from the medical record book by its medical registration number (MRN) because in DHIS database of public hospitals there is no recording site for normal individuals after every diagnosis.

### Data collection tools, techniques and procedures

Data related to that have been confirmed as a type-2 diabetic patient and normal at public hospitals of Afar regional state from the date of database being fully functional (2012) up to data obtaining date (April 22/2020) GC were collected from DHIS database public hospitals. Clients who have been positive for type-2 diabetes were obtained from the database and those who were normal were collected from the medical registration book by medical chart review and used for comparative purposes. The parameters of these samples were collected from their first date of their diagnosis by cross-checking with their start date of their diagnosis to avoid the misleading of machine learning algorithms. From this, the essential variables were collected, therefore, in this study was used to classify and predict type-2 diabete disease status among clients of public hospitals for all ages and both sexes who were diagnosed for diabetes in the region.

### Study variable

#### Dependent variable

The dependent variable is type-2 diabetes disease status with dichotomous response to the question “tested and confirmed as type-2 diabetic patients” if yes = 1″ and “if no = 0”.

#### Independent variables

The above dependent variable was then being modeled to historical predictor variables and reasons that were selected based on existing evidences. The independent variables that have been assessed in this research were:-

- Diastolic Blood Pressure (DBP)
- Systolic Blood Pressure (SBP)
- Fasting Blood Sugar Level (FBS)
- Random Blood Sugar Level(RBS)
- Body Mass Index (BMI)
- Age
- Sex

### Operational definitions

*Accuracy* Accuracy of classifier refers to the ability of classifier to predict the class label correctly, and it also refers to how well a given predictor can guess the value of the predicted attribute for a new data^10^.

*Classification* Is a data mining function that assigns items in a collection to target categories or classes. The goal of classification is to accurately predict (for categorical independent variables) the target class for each case in the data^11^.

*Confusion Matrix* Is a simple performance analysis tool typically used in supervised machine learning. It is used to represent the test result of a prediction model. Each column of the matrix represents the instances in a predicted class, while each row represents the instances in an actual class^12^.

*Kappa Statistics* Are a metric that compares Observed Accuracy with Expected Accuracy (Random chance) that the samples randomly would be expected to reveal^13^.

*Receiver Operating Characteristic test (ROC)* Are a plot of the true positive rate against the false positive rate for the different possible cut points of a diagnostic test. The area under the curve (AUC) is a measure of diagnostic accuracy^8^.

*Prediction* Are a data mining approach that aims at building a prediction (for continuous independent variables) model for classifying new instances into one of the two class values (yes or no)^14^.

*Sensitivity* Is the proportion of positive cases that were correctly identified, which is the most important thing in disease diagnosis^15^.

*Specificity* Is defined as the proportion of negative cases that were classified correctly^16^.

### Data management and quality control

Data related to the outcome variable of type-2 diabetic disease status were selected and extracted from the dataset of DHIS database and from the medical registration book of public hospitals of the region. Furthermore, data cleaning, labeling, coding were done for all selected variables. On the data preprocessing phase, data manipulation and data transformation for incomplete data handling and missing value management were conducted to achieve the best data quality for prediction and classification of type-2 diabetes disease status.

### Data labeling and processing

The raw (preprocessed) data were contained 13 independent; one dependent variable which is categorical variable with 2,239 samples (instances).

Moreover, in this phase, the variable set which had few records (only 2 records) due to its expensiveness, i.e., glycated hemoglobin level, and the other variables which had no role on prediction of type-2 diabetes disease like (MRN and start date), the variables which have similarity among each other (height and weight with BMI) were removed from the final predictor list of variables.

The processed variables were proceeding to data mining activity for prediction and classification of type-2 diabetes disease status and their missing values of each record were replaced with mean value of each variable. Finally, after the data were processed, a total of 2239 clients with their 7 major explanatory variables (attributes) based on variable selection method that were diagnosed for diabetes disease were included for classification and prediction of type-2 diabetic disease status using supervised machine learning algorithms (Table 1).

**Table 1:** Processed diabetes disease status data format from DHIS of public hospitals of Afar in 2021.

Attribute (variable)	Abbreviation	Attribute value	Measurement scale
Sex	S	Male, Female	Nominal
Age	A	number	Numeric
Body mass index	BMI	Number	Numeric
Systolic blood pressure	SBP	Number	Numeric
Diastolic blood pressure	DBP	Number	Numeric
Fasting blood sugar level	FBS	Number	Numeric
Random blood sugar level	RBS	Number	Numeric
Class (outcome variable )	C	Yes, No	Nominal

### Methods of data analysis

Data of clients for diabetes disease status were collected from DHIS database of public hospitals of the region, and then it was checked for its completeness prior to analysis to increase data quality. Data preprocessing was carried out before final analysis to treat missing values and incomplete records. This processed data was changed in to comma separated value (CSV) and attribute relation file format (ARFF) to be loaded in to WEKA-3.7 tool which was used for data analysis purposes. Then the major classification and prediction supervised machine learning algorithms were applied.

On the descriptive part, appropriate descriptive statistical methods such as frequencies, percentages, tables, and graphs were used to summarize and present the findings. For the inferential part, the major supervised machine learning techniques of data mining algorithm for prediction of type-2 diabetes (Logistic regression, ANN, RF, K-NN, SVM, DT pruned J48 and Naïve Bayes) were used. The performance evaluation based on their output for effective prediction and classification of type-2 diabetes disease status among these algorithms was assessed.

### Experimental setup

Since the main objective of this study is applying supervised machine learning algorithms for classifying and predicting new clients’ whether they have type-2 diabetes or not using the information extracted from the diabetes’ dataset. The model building phase in the data mining process of this investigation was carried out under classification and prediction data mining approach. This classification and prediction tasks was conducted using the major supervised machine learning algorithms (DT, pruned J48, artificial neural network, k-nearest neighbor, random forest, Naïve Bayes, support vector machine and Logistic regression) for classifying and predicting the presence or absence of type-2 diabetes disease up on the 70% of the full dataset which was the training data set. To increase classification accuracy, it is better to use many of the dataset for training and few dataset for testing on percentage split of 70 : 30 division^12^.

**Figure 1:**
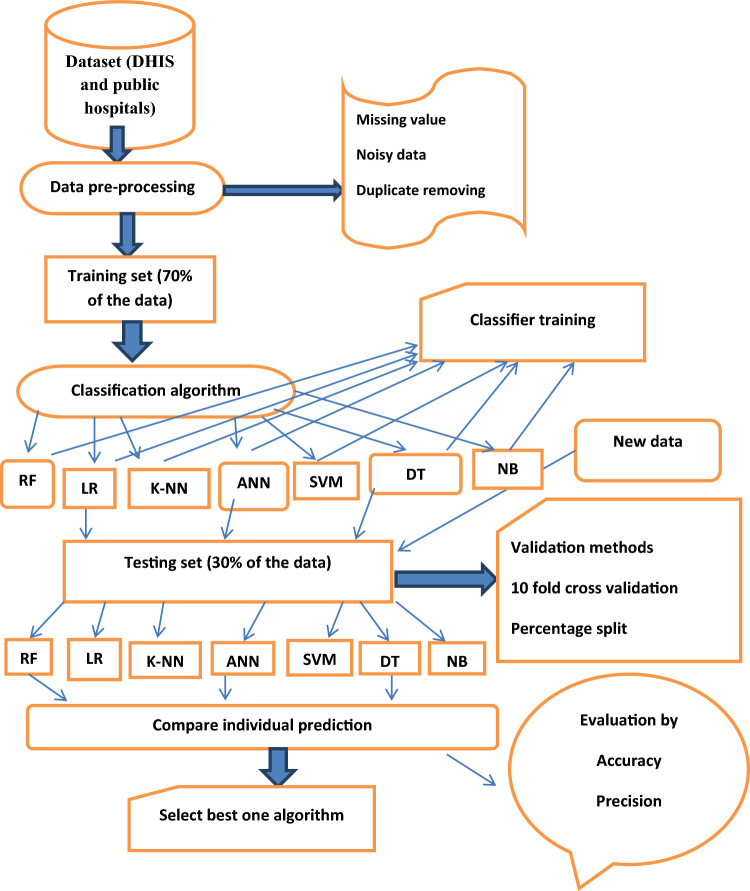
Schematic representation of data mining approach applied using supervised machine learning algorithms for classification and prediction of type-2 diabetic disease status of public hospitals of Afar regional state in 2021.

After that, this task was repeated on the testing dataset, 30% of the full dataset which doesn’t have the target class of type-2 diabetes disease status. Finally, the performance of these supervised machine learning algorithms was evaluated based on their capacity of classification and prediction on the testing dataset^15^. All of these tasks were implemented using WEKA 3.7 data mining tool (Fig. 1).

### Descriptive statistics

Frequency and percentage were used to report categorical variables and mean followed by standard deviation for continuous explanatory variables were used. In addition, confusion matrices were done to show the proportion of different categories of each characteristic with respect to the outcome variable (type-2 diabetes disease status).

### Variable selection method for model building

Commonly used variable selection methods available in commercial software packages, i.e., WEKA tool are Wrapper Subset Forward Selection, Best First -D 1 -N 5 variable selection, and linear forward selection methods. For this study, the Best First -D 1 -N 5 variable selection method was used at tenfold cross-validation and seed number 50 methods which is better for binary outcome studies^17^.

### Model fit statistics

Receiver operating characteristics (ROC) curve was used to assess the general accuracy of the model to the dataset using the area under receiver operating characteristics (AUC). ROC curve is a commonly used measure for summarizing the discriminatory ability of a binary prediction model.

The ROC curve describes the relationship between sensitivity and specificity of the classifier. Since the ROC curve cannot quantitatively evaluate the classifiers, AUC is usually adopted as the evaluation index. AUC value refers to the area under the ROC curve. An ideal classification model has an AUC value of 1, with a value between 0.5 and 1.0, and the larger AUC represents that the classification model has better performance^18^.

### Model diagnostics

The performance evaluation (model diagnosis) of different supervised machine learning techniques of data mining algorithms for prediction and classification of type-2 diabetes were carried out. This was being done using cross-validation and different confusion matrices.

Cross-validation is a technique used to evaluate the performance of classification algorithms. It is used to evaluate error rate for learning techniques. The dataset is portioned in to n-folds; each fold is used for testing and training purposes. The procedure repeats for n times in testing and training dataset. In a tenfold cross validation the data is divided in to 10 parts where each part is approximately the same to form the full dataset. Each term is held out and during the learning scheme which trained on the remaining nine-tenths, the error rate is calculated in the holdout set. Learning procedure executes 10 times on training sets and finally the error rates for 10 sets are averages to yield an overall error rate^10^. A confusion matrix is used to present the accuracy of classifiers obtained through classification. It is used to show the relationship between outcomes and predicted classes (Table 2).

**Table 2:** Different outcome of two-class prediction used for performance evaluation.

	Predicted as “yes”	Predicted as “no”
Actual “yes”	True positive “TP”	False negative “FN”
Actual “no”	False positive “FP”	True negative “TN”

In addition to the confusion matrices, there are also different parameters used to compare the performance of supervised machine learning algorithms for their classification and prediction capacity. The table below contains performance comparison matrices with their respective formulas (Table 3).

**Table 3:** Performance evaluation matrix for the classification and prediction model with their formula.

Performance metrics	Formula
Recall/sensitivity	TP/(TP + FN) *100
Specificity/true negative rate	TN/(TN + FP) *100
Correctly classified instances (CCI); accuracy	TP + TN/(TN + FN + TP + TN)*100
Incorrectly classified instances (ICI)	FP + FN/(TN + FN + TP + TN)*100
Kappa statistics	Observed accuracy—expected accuracy/1-expected accuracy

Source:^4^.

Each model which was used for classification and prediction algorithm was diagnosed based on their accuracy. Since this study was a medical database record review design which is having known class (confirmed as type-2 diabetic patient or not), then we were used for the supervised data mining techniques. The machine learning algorithms and model specifications for prediction and classification of diabetes disease were focused on high performance algorithm and from high dimensional medical dataset. These model comparisons were conducted according to the following table format (Table 4).

**Table 4:** Criterion’s used for Performance comparison of applied supervised machine learning algorithms used for classification and prediction of type-2 diabetes disease status in Afar regional state.

Classifier	Measurements					
	Accuracy (CCI)	Sensitivity	Specificity	ROC	Kappa statistics	Confusion matrix
Decision tree (J48 pruned)						
ANN(multilayer perceptron)						
K-NN (Lazy)						
Random forest						
Naïve Bayes						
Logistic regression						
SVM (RBF Kernel)						

### Model specification

Classification models have been used to determine categorical class labels, meanwhile prediction models were used to predict continuous functions, this was done in the following steps:- Data cleaning, Relevance analysis, and Data Transformation. The obtained datasets was preprocessed and split into 2 sets, training (70% of the total dataset) and test data (30% of the dataset) (13, 15). The following models were specified for data mining in classification and prediction of type-2 diabetes disease status accordingly.

### Ethics approval and consent to participate

The study was approved by the Institutional Review Board of Samara University. A letter of support was obtained from Samara University. All results of this research were based on the use of secondary data and the data collection was performed prospectively. Therefore, an informed written consent form from the public hospital DHIS Database coordinator was obtained and the study was conducted in accordance with the ethical standards of the institutional and national research committee.

## Results

### Descriptive statistics

The descriptive statistics of the dataset (mean, maximum, minimum, and standard deviation of continuous variables) are presented in Table 5 based on the WEKA tool of attribute selection Best First -D 1 -N 5method.

**Table 5:** Results of variable selection method with their minimum, maximum, mean, and std. deviation values of diabetes disease status dataset of DHIS of public hospitals of Afar region 2021.

Parameter	Minimum	Maximum	Mean	Std. Deviation
Sex	–	–	–	–
Age	16	92	48	15.8
BMI	11	35	23.2	4.2
SBP	80	173	124	19
DBP	55	95	76.1	8.5
FBS	65	209	128.5	27.3
RBS	89	420	257	43

**Figure 2:**
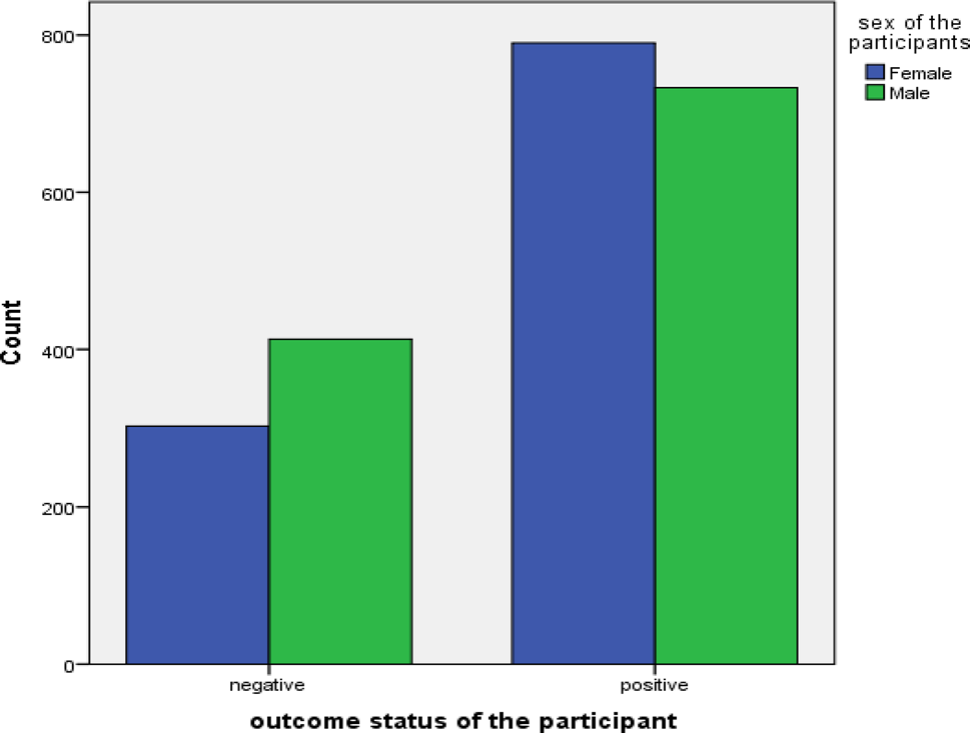
Proportion of total participants in terms of sex and type-2 diabetes disease status of DHIS diabetes dataset in 2021.

In addition, the following clustered bar chart stated that out of 2239 total participants, almost half of them 1146 (51.1%) were male represented by green color, 1093 (48.9) were female represented by blue color and 1523 (68%) were positive for type-2 diabetes where as 716 (32%) of them were negative (Fig. 2).

**Figure 3:**
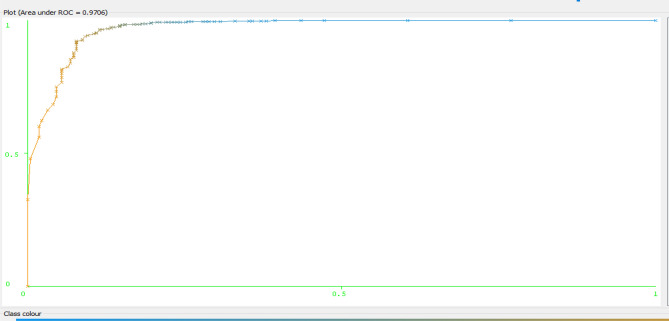
Area under the ROC curve of random forest used for classification and prediction of type-2 diabetic disease status of Afar region.

The graphical output of WEKA illustrated below revealed that the descriptive statistics of total participant’s for type-2 diabetes disease status in the study area. From 2239 of the total sample, 1523 of them were positive for type-2 diabetes and represented in blue color, whereas 716 of the sample were negative and represented in red color. In addition, the Age, Sex, FBS, RBS, DBP, and SBP of the participants are also illustrated in this graphical output (Fig. 3).

### Inferential statistics

From the total dataset 1567 (70%) of the sample was used as a training dataset of the supervised machine learning algorithms and 672 (30%) of the dataset was used as a testing dataset. This classification was conducted at tenfold cross-validation to prepare training and testing dataset. In this study, seven supervised machine learning algorithms were implemented for classification and prediction of type-2 diabetic disease status.

From the seven supervised machine learning algorithms, the highest classification and prediction capacity was obtained from RF algorithm at 93.8% of CCI, followed by DT pruned J 48 and K-NN algorithm at 91.8%% and 89.8% of CCI rate respectively. The lowest prediction and classification capacity was obtained from SVM (RBF) algorithm at 85.5% of correctly classification instance rate (Table 6).

**Table 6:** Performance evaluation of supervised machine learning algorithms implemented for classification and prediction of type-2 diabetic disease status in Afar region.

Machine learning Algorithms used	Evaluation criteria for classification Algorithm implementation			
	Time taken to build the model	CCI (%)	ICI (%)	Kappa statistics
DT (J48 pruned)	0.11 s	91.8	8.2	0.80
NB	0.02 s	86	14	0.68
ANN	4.25 s	88.8	20.2	0.74
K-NN (Lazy)	0.01 s	89.8	10.2	0.76
SVM (RBF)	0.2 s	85.5	14.5	0.66
RF	1.48 s	93.8	6.2	0.85
Logistic regression	1.62 s	87.2	12.3	0.69

The other evaluation criteria for classification Algorithm Kappa Statistic were implemented for numeric value only. To choose the best algorithms for soaring performance; different algorithms were implemented and evaluated with respect to some evaluation criterion on the selected dataset. From the table sited below, the kappa statistics of RF algorithm showed that the best result (0.85) followed by DT pruned J48 (0.80) and K-NN (0.76) (Table 6).

DT pruned J48 algorithm was implemented for generating pruned tree. The tree generated by J48 can be used for classification of whether a client has tested positive or negative for type-2 diabetes. This data mining technique uses the concept of information gain. Each attribute of the data is used to decide by splitting the data into smaller modules.

The figure stated below showed that a pruned J48 DT which classifies the type-2 diabetes disease status of the test dataset at 18 number of leaves and 35 size of tree. Furthermore, this method has been reducing misclassification of data which often occurs in learning process. Pruning methods have been introduced to reduce the complexity of tree structure without decreasing classification and prediction accuracy.

For better understanding, results for each supervised machine learning algorithms of data mining technique have shown in different tables. Accordingly, the efficiency of the supervised machine learning classifier and predictors can be assessed with numerous measures. The estimate of these measures depends on the contingency table, which is further obtained from the classification and prediction algorithms which were implemented.

The classification and prediction algorithm which achieves the best performance in provision of soaring specificity and sensitivity value is measured by the finest algorithm. From the table stated below, it is clear that the RF and DT pruned J48 algorithms achieved the maximum values (specificity = 0.93, sensitivity = 0.98, and ROC curve = 0.97) and (specificity = 0.91, sensitivity = 0.96 and ROC curve = 0.91). This performance is followed by K-NN with 0.92% sensitivity and specificity (Table 7).

**Table 7:** Specificity, sensitivity, and ROC curve used for comparison of accuracy measures of each classifier implemented for classification and prediction of type-2 diabetic disease status of Afar region.

Supervised machine learning algorithms implemented	Accuracy measures used for performance comparison			
	Precision (specificity) %	Recall (sensitivity) %	ROC	Class to be predicted
DT (pruned J48)	0.91	0.96	0.91	+ve (1)
	0.91	0.82	0.91	−ve (0)
NB	0.89	0.89	0.88	+ve (1)
	0.78	0.78	0.88	−ve (0)
ANN	0.89	0.92	0.90	+ve (1)
	0.90	0.76	0.90	−ve (0)
K-NN (Lazy)	0.92	0.92	0.88	+ve (1)
	0.84	0.83	0.88	−ve (0)
SVM (RBF)	0.87	0.91	0.88	+ve (1)
	0.80	0.73	0.88	−ve (0)
RF	0.93	0.98	0.97	+ ve (1)
	0.95	0.84	0.97	−ve (0)
LR	0.88	0.93	0.90	+ ve (1)
	0.84	0.74	0.90	−ve (0)

The figure stated below showed that the area under the ROC curve of random forest (RF) which was 0.97, which is the largest value with excellent performance. The ROC curve describes the relationship between sensitivity and specificity of the classifier. Since the ROC curve cannot quantitatively evaluate the classifiers, AUC is usually adopted as the evaluation index. AUC (area under ROC curve) value refers to the area under the ROC curve. An ideal classification model has an AUC value of 1, with a value between 0.5 and 1.0, and the larger AUC represents that the classification model has better performance (Fig. 3).

The performance of any classification algorithm is extremely depending on the nature of the training dataset used. In WEKA tool, confusion matrices which are generated after simulation of classification and prediction algorithms are very constructive for evaluating classifiers. The columns in the confusion matrix represent the predicted classification class, and the rows represent the actual classes.

The table stated below showed that the confusion matrices of each classifier used tenfold cross-validations. From this, RF and DT pruned J48 algorithm showed the best prediction performance for tested positive instances out of 454 actual positive individuals, 446 and 438 of them had predict as tested positive, respectively. These prediction performances were followed by ANN and K-NN algorithms at 437 and 421 instances predicted as tested positive for type-2 diabetes out of 454 actual positive testing dataset participants (Table 8).

**Table 8:** Confusion matrices of each classifier used at tenfold cross-validation used for classification and prediction of type-2 diabetic disease status of Afar region.

Classification algorithm implemented	Accuracy measures used for performance comparison		
	Tested positive	Tested negative	Class to be predicted
DT (pruned J48)	438	16	1
	39	179	0
NB	407	47	1
	47	171	0
ANN	417	37	1
	51	167	0
K-NN (Lazy)	421	33	1
	35	183	0
SVM (RBF)	414	40	1
	57	161	0
RF	446	8	1
	33	185	0
LR	424	30	1
	56	162	0

## Discussion

The study identified the prediction and classification accuracy of RF [correctly classified rate (93.8%), kappa statistics (0.85), sensitivity (0.98), AUC (0.97) and confusion matrix (out of 454 actual positive predict as positive for 446 of them)], DT pruned J48 [correctly classified rate (91.8%), kappa statistics (0.80), sensitivity (0.96), AUC (0.91) and confusion matrices (out of 454 actual positive predict as positive for 438 of them)],K-NN [correctly classified rate (89.8%), kappa statistics (0.76), sensitivity (0.92), AUC (0.88) and confusion matrices (out of 454 actual positive predict as positive for 421 of them)].

Furthermore, prediction and classification accuracy were obtained from SVM [correctly classified rate (85.5%), kappa statistics (0.66), sensitivity (0.91), AUC (0.88) and confusion matrix (out of 454 actual positive predict as positive for 414 of them)], logistic regression [correctly classified rate (87.2%), kappa statistics (0.69), sensitivity (0.93), AUC (0.90) and confusion matrices (out of 454 actual positive predict as positive for 424 of them)], ANN [correctly classified rate (88.8%), kappa statistics (0.74), sensitivity (0.92), AUC (0.90) and confusion matrices (out of 454 actual positive predict as positive for 417 of them)] and naïve Bayes [correctly classified rate (86%), kappa statistics (0.68), sensitivity (0.89), AUC (0.88) and confusion matrices (out of 454 actual positive predict as positive for 407 of them)].

From the seven major supervised machine learning algorithms, the best classification and prediction result for type-2 diabetes disease status was obtained from RF classification and prediction algorithm 93.8%ofcorrectly classification capacity. In the WEKA tool, the percentage of accurately classified instances is called the accuracy of the classifying model. RF algorithm also shows an excellent prediction capacity at 100% accuracy on other studies conducted by Tejashri and his colleagues in 2015 at Wagholi, Pune, India^19^ and the study conducted by Naqvi and his colleagues in 2018 found that RF (accuracy of 89.3%)as the best technique for prediction of diabetes^20^.

On the other hand, the best accuracy of this study obtained from random forest (RF) (93.8%) is higher than the accuracy which was obtained from the previous study (85.9%) by Iyer and his collageus. This might be due to the number of input variables used because our study used seven input variables while the other study used only four variables (Plasma glucose concentration, BMI, DPF, and Age) as an input variable for classification and prediction of diabetes disease status^13^.

Based on their accuracy, this algorithm (RF) was followed by DT-pr-une-d J48 and k-nearest neighbor (K-NN) algorithms at 91.8% and 89.8%, respectively. Another study carried out by Roobini and Lakshmi on 2018 showed that DT pruned J48 and K-NN accuracy of 91.72% and 91.14% respectively, which is consistent in accuracy with our research study^3^.

Basically Pruned J48 Tree is fast DT learning and it builds a DT based on the information gain or reducing the variance. It examines normalized information gain (difference in entropy) that results from choosing an attribute as a split point. The highest normalized IG is used at the root of the tree. The DT pruned J48 generate a tree contained 18 number of leaves with 35 size of tree that gets an accuracy of 91.8%. This help to extract certain rules and reduce the risk of over fitting on the training data. From this, variable age was obtained as a root node with the highest information gain (870.0/148.0) when age is >= 36 year to be more likely tested positive for type-2 diabetes others as a leaves. This result is consistent with the result obtained from a study conducted by Amatul and his colleagues in 2017 from Indian diabetes dataset for prediction and classification of type-2 diabetes disease status which showed a diagnostic result of positive for type-2 diabetes disease was at the age of 40 years old^21^.

In addition, other rules which can be extracted from this schematic representation showed that age >= 36 years which is the root node, DBP > 81, FBSL > 115, RBSL > 211, sex = female and BMI > 25 more likely to be predicted as tested positive for type 2 diabetes. Another study entitled as “Diagnosis of diabetes using classification mining techniques” which was conducted by Aiswarya and his colleagues^22^ showed that plasma glucose concentration as a root node. This difference may be due to this study considers both fasting and random glucose levels separately but the other study used in combination as a single variable.

The other alternatives that have been used in this study for performance comparison were kappa statistics. From this kappa statistics that compares observed accuracy with expected accuracy (random chance) out of the seven supervised machine learning algorithms, it was higher in RF algorithm which was 0.85 and followed by DT pruned J48 (0.80) and K-NN (0.76).The value of kappa statistics of the second best algorithms (DT = 0.80) is almost double of the value of kappa statistics of another study DT J48 algorithm which was 0.47^13^. This inconsistent result might be due to the effect of pruning because pruning reduces the occurrence of events due to chance.

The study conducted at United Arab Emirates in 2015 was found the higher kappa statistics result from naïve Bayes algorithm 0.50 followed by J48 DT which was 0.47^15^. Normally, the value of kappa statistics ranges from − 1 up to + 1, meaning the value 1 indicates perfect classification -1 indicates wrong classification, i.e., all the classifications are occurred due to chance. Based on this range, the result of this study obtained from random forest, DT, pruned J48, and K-NN for classification of type-2 diabetes disease status lies on excellent classification and prediction capacity.

As can be seen from the result table of Table 7, in this study, the performance of the seven supervised machine learning algorithms was compared to their classification performance based on specificity, sensitivity, and area under ROC curve (Table 7). For the testing dataset, the comparative analysis results demonstrated that the random forest DT pruned J48 and K-NN algorithms showed the best result of sensitivity of 98%, 96%and 92% respectively. On the base of specificity, the best result was observed in random forest, K-NN, and DT pruned J48 algorithms with 93%, 92%, and 91%, respectively. On the other hand, their area under the ROC curve showed that almost similar results ranging from (0.88–0.91) except the maximum ROC curve result obtained from random forest which is 0.97. Surprisingly, in all aspects of performance comparison criteria, random forest algorithm came out to be the best algorithm with a classification and prediction capacity based on its excellent performance of sensitivity, specificity, and ROC curve. A similar result was obtained from another study conducted at Pakistan in 2018 by Naqvi and his colleagues with the highest sensitivity of random forest at 96.2%^20^.

In addition, a review paper reviewed by Chui and his colleagues in 2017 showed that RF algorithm had an excellent capacity on classification and prediction of type-2 diabetes at a ROC curve of 0.98, which was, exactly similar with our study^23^.

Another research work conducted on Classification of Diabetes patient by using Data Mining Techniques in 2018 by Nidhi and his colleagues^24^ showed that a better classification performance by DT J48 at 65.3% of sensitivity. This result is even lower than the true sensitivity of our result obtained from DT J48 algorithm which was 96% of sensitivity. This difference in prediction and classification performance may be due to the difference in sample size used for data mining and the attributes used to classify and predict type-2 diabetes disease status. In the previous study, they have been conducted on 768 instances with eight attributes where as in our research study we used 2239 samples with eight attributes. This indicated that an increase in sample size will lead to better classification and prediction capability of these supervised machine learning algorithms’^11^.

From the result of confusion matrix showed on Table 8 which was conducted on 672 samples of testing dataset contained 454 actual positive and 218 actual negative for type-2 diabetes. On the basis of these evaluation criteria, the seven supervised machine learning algorithms, random forest and DT pruned J48 algorithms were revealed the highest prediction capacity on forecasting of true positive, i.e. out of 454 actual positive samples they predict 446 and 438 of them as positive respectively. On the other hand, the lowest prediction performance was obtained from the SVM algorithm out of 454 actual positive participants, it predicts to be tested positive only for 414 of them. This study result is consistent with the study conducted in Wagholi, Pune, India, in 2015 entitled as Data Mining Approach for Diagnosing Type-2 Diabetes status^19^.

### Strength and limitation of the study

This study was carried out using an appropriate methodological approach with sufficient amount of sample size for data mining. Methodologically, the study covered seven supervised machine learning algorithms (ANN, K-NN, Naïve Bayes, DT, pruned J48, logistic regression, RF, and SVM). The best three classifier and predictor models were selected for prediction of the upcoming new client for type-2 diabetes diagnosis. As a result, these points are considered as the strength of the study.

On the other hand, this study was having the following limitations: firstly, since the study was conducted on secondary data, some of the important variables for classification and prediction of type-2 diabetic disease like glycated hemoglobin level were missed. The reason behind the removal of this important predictor variable is the occurrence of very few (only 2) diagnostic results recorded for this variable due to its expensiveness to perform the test. Secondly, while some of the record values of certain instances are missed; has been replaced with mean which affects the accuracy of prediction and classification performance, so that this also considered as limitation.

## Conclusion and recommendations

In this study, seven supervised machine learning algorithms were implemented; compare their performance of classification and prediction for type-2 diabetes disease status from DHIS database at public hospitals of Afar region in 2021 for better decision making. Supervised machine learning algorithm is one of the essential aspects of hidden and domain-specific knowledge extraction to facilitate diagnostic activity for rapid and suggestive diagnosis and early intervention in the study area in particular and future generation in general.

In this research study, we are considered the dataset of diabetes patients which is further collected at DHIS database of public hospital of Afar region. The dataset has 2239 instances with eight different selected attributes. In this study, seven major supervised machine learning algorithms (DT, pruned J48, K-NN, Logistic regression, SVM, ANN, Naive Bayes, and RF) were simulated and found that the random forest, DT pruned J48 and K-NN have the maximum accuracy (93.8%, 91.8% and 89.8%) respectively for classifying and predicting type-2 diabetes disease whether they are tested positive or tested negative.

The constructed model could assist healthcare providers to make better clinical decisions for type-2 diabetes disease diagnosis. Additionally, the model could be further developed for patient protection through rapid diagnosis and early intervention to avoid further complications occurred due to type-2 diabetes. In the future, the results can be utilized to create a control plan for type-2 diabetes patients because diabetes patients are normally not identified until a later stage of the disease or the development of complications.

Therefore, based on the above finding and conclusion, the following recommendations are forwarded:

- Random forest supervised machine learning algorithm can be implemented for facilitating the diagnostic activity and early intervention.
- These results findings might be utilized to create a control plan for type-2 diabetes to prevent development of complications.
- The techniques used may be helpful to clinicians to develop an accurate and effective tool to make a better and suggestive decisions about type-2 diabete disease status of clients.
- Further study should be conducted based on primary data and by incorporating other predictor variables which were not considered in this study to detect the exact accuracy of these machine learning algorithms.

## Data Availability

The datasets supporting the conclusions of the study are included in the article. Any additional data will be available on request. The datasets used and/or analyzed during the current study are available from the corresponding author on reasonable request.

## Abbreviations

ANN: Artificial neural network
ARFF: Attribute relation file format
AUC: Area under the curve
BMI: Body mass index
CCI: Correctly classified instances
CSV: Coma separated value
DHIS: Demographic health information system
HMIS: Health information management system
ICCI: Incorrectly classified instances
KDD: Knowledge discovery in database
KNN: K- nearest neighbor
WEKA: Waikato Environment for Knowledge Analysis
